# Shaping the Future of Infectious Diseases: The Journey to Promote Value, Opportunity, and Positive Messaging

**DOI:** 10.1093/ofid/ofae669

**Published:** 2024-11-12

**Authors:** Molly L Paras, Lisa M Chirch, Mariam Aziz, Gayle P Balba, Constance Benson, Saira Butt, Scott H James, Todd P McCarty, Raymund Razonable, Rebecca Reece, Rachel Shnekendorf, Talia H Swartz, J Alex Viehman, Vera P Luther, For the Infectious Diseases Society of America Fellowship Training Program Directors Community of Practice and Working Group (complete list of contributing authors in the Working Group listed in acknowledgments), Emily Abdoler, Kartikey Acharya, Michael Angarone, Jennifer Babik, Rachel Bartash, Nitin Bhanot, Brian Blackburn, Emily Blumberg, Dana M Blyth, Daniel Bourque, Andres Bran, Victoria Burke, Adrienne L Carey, Laura L Cheney, Brian D Chow, Lisa A Clough, Cheston Cunha, Jorgelina T de Sanctis, David M Dobrzynski, Ige A George, Melanie Goebel, Eli S Goshorn, Ramiro Gutierrez, Erica S Herc, Molly J Hillenbrand, Anna Kaltsas, Sarwat Khalil, John Kiley, Dora A Lebron, Mikyung Lee, Anne-Marie Leuck, Raul Macias Gil, Christopher Mapa, Luis A Marcos, Brionna Matt, Eileen K Maziarz, Michael Melia, Subhashis Mitra, Lea Monday, Brian Montague, Holly A Murphy, Elizabeth E Novick, Obinna N Nnedu, Priya Nori, Sharon Ongunti, Georgina Osorio, Rosalie Pepe, Federico Perez, Edward F Pilkington, Jillian E Raybould, Gail E Reid, Sara Robinson, Martha Sanchez, Sara Schultz, Christopher Sellers, Matthew Simon, Lauren Sisco, Magdalena Slosar-Cheah, Mohammad Mahdee E Sobhanie, Ann Stapleton, Wendy Stead, Judy Streit, Deborah A Theodore, Noah Wald-Dickler, Devin M Weber, Scott A Weisenberg, Kelsey L Witherspoon, Joseph M Yabes, Richard A Zuckerman

**Affiliations:** Division of Infectious Diseases, Massachusetts General Hospital, Harvard Medical School, Boston, Massachusetts, USA; Division of Infectious Disease, University of Connecticut School of Medicine, Farmington, Connecticut, USA; Divisions of Infectious Diseases and Community and Global Health Equity, Rush University Medical Center, Chicago, Illinois, USA; Division of Infectious Diseases, Medstar Georgetown University Hospital, Georgetown University School of Medicine, Washington, District of Columbia, USA; Division of Infectious Diseases and Global Public Health, University of California San Diego, San Diego, California, USA; Division of Infectious Diseases, Indiana University School of Medicine, Indianapolis, Indiana, USA; Division of Pediatric Infectious Diseases, University of Alabama at Birmingham, Birmingham, Alabama, USA; Division of Infectious Diseases, University of Alabama at Birmingham, and Birmingham Veterans Affairs Medical Center, Birmingham, Alabama, USA; Division of Infectious Diseases, Mayo Clinic College of Medicine and Science, Rochester, Minnesota, USA; Division of Infectious Diseases, West Virginia University School of Medicine, Morgantown, West Virginia, USA; Infectious Diseases Society of America, Arlington, Virginia, USA; Division of Infectious Diseases, Icahn School of Medicine at Mount Sinai, New York, New York, USA; Division of Infectious Diseases, University of Pittsburgh, Pittsburgh, Pennsylvania, USA; Section on Infectious Diseases, Wake Forest University School of Medicine, Winston-Salem, North Carolina, USA

**Keywords:** infectious diseases fellowship, medical education, positive messaging, value

## Abstract

The field of infectious diseases (ID) offers a rewarding career path and is widely viewed as an essential subspecialty in medicine. However, in recent years, these positive aspects have been overshadowed by concerns surrounding low fellowship match rates, undercompensation, and burnout. The Infectious Diseases Society of America Fellowship Training Program Directors Committee met in 2023, discussed the future of ID as a specialty, and sought to develop strategies to highlight the value and opportunities of ID for future generations, as well as underscore the importance of and provide tools for positive messaging to trainees about the subspecialty. This paper presents ideas generated at this meeting and is meant to serve as a reference for ID training program directors, as well as the wider ID community, in uplifting and shaping the future of the field.

## Teaser

To preserve and grow the infectious diseases (ID) workforce, ID professionals should be intentional in using positive messaging to highlight the specialties' value and opportunities, while promoting programs and policies that foster a resilient ID community.

The field of infectious diseases (ID) offers a meaningful and rewarding career path and is an essential subspecialty in medicine. Despite many positive aspects, concerns about low fellowship match rates, undercompensation, and burnout have overshadowed the field's appeal [1, 2]. Calls to rebrand and consider reasons for optimism emerged in the interest of preserving and growing the workforce [3].

The annual IDWeek National Fellowship Training Program Director (PD) meeting allows PDs and associate PDs to discuss contemporary topics relevant to fellowship training. Because of national conversations in published literature and on social media on the topics of fellowship recruitment and concerns related to the workforce pipeline, members of the Infectious Diseases Society of America (IDSA) Fellowship Training Program Directors Committee (PDC) selected “Preserving the Future of ID” as the theme of the 2023 IDWeek meeting. After listening to speakers with content expertise on the subtopics of value, opportunity, and positive messaging, attendees participated in small group discussions moderated by PDC members. Meeting notes captured the conversation and ideas generated and served as a foundation for this manuscript. Attendees of the meeting were invited via email to participate in writing this manuscript. Members of the PDC, as well as PD and associate PD volunteers, drafted individual sections that were ultimately combined. All coauthors reviewed and approved the complete manuscript.

## VALUE

Identifying and recognizing the value that ID professionals and services add to institutions, medical education, patient care, and public health is foundational to shaping the future of the subspecialty [4]. ID professional services have been underrecognized, undervalued, and undercompensated in today's healthcare and medical educational systems [1]. Redefining, measuring, and acknowledging the value of ID professionals and services more effectively can reverse these trends and shape the future of the subspecialty.

Table 1 summarizes areas where the value of ID is demonstrable and measurable. In addition to those highlighted, it is essential to address and mitigate the high cost of medical education, which often deters trainees from pursuing ID careers. Providing financial literacy education helps fellows manage their earnings, advocate for fair compensation, and highlight the value of their skills and expertise. Fellowship programs should develop leadership skills and tools during training to enhance value recognition. ID physicians’ expertise in managing complex and multisystem diseases makes them invaluable resources to institutions and public health programs. Training programs that emphasize these skills and encourage ID physicians to pursue leadership roles in hospital, healthcare, and public health organizations will ensure ID remains central in strategic decision-making. Importantly, using those leadership skills to redefine compensation systems will be critical. The movement toward increased non–relative value units-based ID physician compensation for uncompensated work, such as surgical comanagement agreements and cost saving interventions, would benefit both the healthcare system and ID workforce [8, 9].

**Table 1.** Value Added of Infectious Diseases Professionals and Services

ID Value Domains	Examples of Value Added by ID Professionals/Services
**Adding value to institution/hospital care**	Infection prevention and control services (eg, outbreak investigations, contact tracing, implementing pathogen-based infection prevention and control policies)
…	Antimicrobial stewardship services (eg, decreased length of stay, cost-savings)[5]
…	Microbiology laboratory management (eg, appropriate diagnostic tests, evaluation of new/novel technologies, hospital pathogen surveillance)
…	OPAT management (eg, decreased length of stay, antimicrobial adverse effect monitoring)[6]
…	Multidisciplinary team contributions of ID professionals (ie, transplant, orthopedics, cardiovascular, ICU/critical care, substance use disorder)
**Adding value to patient care and improving patient outcomes**	Inpatient ID consultation (eg, improved outcomes in patients with *Staphylococcus aureus* bacteremia)[7]
…	Outpatient clinic management of acute and chronic infectious diseases
…	Infection prevention and control services
…	Antimicrobial stewardship services (eg, appropriate antibiotic selection and duration, reduced mortality rates)[5]
…	Microbiology laboratory management
…	OPAT management (eg, demonstrated safe, clinically effective care)[6]
…	Multidisciplinary team contributions of ID professionals
**Adding value to the educational mission (medical school, hospital, outpatient)**	Medical school course instruction in microbiology and infectious diseases
…	Inpatient and outpatient instruction of medical students, residents, fellows, ancillary team members
…	Educating allied healthcare professionals (eg, nurse practitioners, physicians’ assistants, pharmacists, other advanced practitioners)
…	Faculty mentoring (eg, ID research, professional development, education)
…	Peer education (eg, grand rounds, ID subspecialty rounds, shared patient care discussions)
**Adding value to society and public health**	Community education (eg, community programs, media)
…	Public health departments (eg, pandemic response, STI, HIV, TB, hepatitis, migrant and refugee health programs)
…	Societal impact of contributions to hospital epidemiology/infection prevention and control (eg, manage outbreaks, reducing nosocomial infection rates, reducing antimicrobial resistance rates and inappropriate antibiotic usage in the community)
**…**	Telemedicine (eg, provide subspeciality care in resource-limited settings)

Abbreviations: ICU, intensive care unit; ID, infectious diseases; OPAT, outpatient parenteral antimicrobial therapy; STI, sexually transmitted infection; TB, tuberculosis.

## OPPORTUNITY

Providing trainees with opportunities and experiences to showcase the vast number of career pathways in the field is critical to shaping the future of ID. ID physicians should foster, mentor, and sponsor learners’ engagement early in training, including the premedical school stage, to inspire and influence learners in their formative years [10–12]. ID specialists should also align values and interests important to younger generations through programs highlighting the role and impact of ID linked to health disparities, social justice, substance use disorders, and climate change, among others, to optimize engagement [13, 14]. Specific examples of engagement opportunities are included in Table 2.

**Table 2.** Key Components of Promoting Infectious Diseases as a Specialty Through Opportunities and Positive Messaging

Component	Area of Interest	Actionable Activity
**Engage, inspire, and connect with future members**	Precollege and undergraduate student education	Promote uniform positive message of ID as a prestigious field with exposure to multiple areas in healthcare and diversity of roles and job opportunities through personal connections, program websites, and social media platforms
	Medical student education	Mentorship and role modeling experiences with faculty–student liaisons including through nontraditional opportunities such as community health fairs, “medical scribe” positions in ID clinic
	…	School or site visits/open house activities
	…	Teaching in early medical school didactic curriculum and clinical electives; emphasis on the rich diversity of clinical practice and role as advocates for patients; less emphasis on rote memorization
	Medical resident education	Student and resident electives and shadowing activities (inpatient consult service, outpatient, infection prevention and control teams, laboratory electives)
	…	Involvement in scholarly activity/quality Improvement/antimicrobial and diagnostic stewardship projects including in summer undergraduate research programs as well as IDSA-sponsored programs such as the Grants for Emerging Researchers/Clinicians Mentorship Program and iDMentorship 365 Program
	…	Support for ID interest groups and social/educational activities including in local/regional ID conferences and societies
**Highlight the critical role of the specialty**	Clinical practice	Curing and controlling infections
	…	Vaccines as prevention
	…	Health equity
	Research	Promoting research in emerging areas of ID
	…	Antimicrobial drug discovery
	…	Vaccine development
	Medical education	Exhibiting excellence in teaching; serving in medical education leadership roles (eg, program directors, deans)
	…	Leading the preparation and navigation of future pandemics
	…	Leading infection prevention and control efforts
	Leadership	Promoting work at national level with Centers for Disease Control and Prevention
	…	Promote policy and advocacy in public health/global health
**Highlight unique and satisfying aspects of ID careers**	Spectrum of practice and variety of practice models	Clinical, research, public health, consulting, biomedical/industry job opportunities
	…	Longitudinal patient care
	…	Short-term/limited patient care when cure established
	…	Job flexibility (outpatient practice, inpatient practice, telehealth)
	…	Centers for Medicare & Medicaid and The Joint Commission's mandate of infection prevention and control and antimicrobial stewardship programs
	Job security and evolving needs of expertise	Emerging and reemerging infections
	…	Impact of societal events (eg, climate change, human conflict and migration)
	…	Incorporation of artificial intelligence into clinical and research practice
**Promote member representation and diversity of members**	Race and ethnic diversity	Develop community partnerships
	Age diversity	Promote diversity and inclusiveness in the society
	Gender diversity	Ensure equitable opportunities for all members
	Geographic diversity	Recognition and promotion of members at the local (hospital and community), state, and national level
**Emphasize diverse opportunities**	Private or academic practice	Student and resident electives and shadowing activities
	Hospital and outpatient practice	…
	Clinical, translation and basic science research	Summer undergraduate research programs
	Public health	…
	Industry	…
	Leadership opportunities	…
**Celebrate accomplishments**	Pandemic preparedness	Rapid deployment of clinical trials, protocols and guidelines (eg, COVID-19 pandemic)
	…	Outbreak investigations
	Infection prevention and control	…
	Clinical cure of infections	Patient satisfaction with prevention and cure (eg, U = U initiative)
	Vaccination	Prevention of infections (eg, eradication of smallpox, rapid development of mRNA platform during COVID-19 pandemic)
	Antimicrobial drug discovery and clinical development	Novel drugs for drug-resistant pathogens
	Publishing personal stories and highlighting distinguished careers	Written narrative stories in high-profile journals
**Offer solutions to issues**	Individual level	Promote work–life balance
	…	Seek mentorship
	Hospital level	Compensate for nonclinical work
	…	Protected time for career development and advancement
	…	Advocate for fair reimbursement and loan repayment (eg, NIH Loan Repayment Programs, HELP Act, Bio-Preparedness Workforce Pilot Program)
	Society level	…

Abbreviations: HELP Act, HIV Epidemic Loan-Repayment Program Act; ID, infectious diseases; IDSA, Infectious Diseases Society of America; NIH: National Institutes of Health; U = U, undetectable equals untransmittable.

With a significant emphasis on the inpatient training in fellowship, trainees have minimal exposure to the broader career opportunities possible, including academic medicine, private practice, public and global health, and industry. There are expanding opportunities in telemedicine and other positions that provide job flexibility and the opportunity to serve in unique practice environments [15]. The need for future antimicrobials means ID physicians are necessary to guide the development of new agents and bring them to market sensibly and meaningfully by working within and alongside pharmaceutical companies. Within these career paths, ID physicians hold critical leadership roles and affect meaningful change as leaders of quality improvement and population-based initiatives such as Infection Prevention and Control and Antimicrobial Stewardship. On a global scale, the critical role of the ID specialty in pandemic preparedness and response, combating multidrug-resistant organisms, leading global health initiatives, and pioneering a holistic approach to addressing social determinants of health should be emphasized. Many fellowship programs have adapted to these diverse career paths by creating elective rotations and tracks to further prepare their graduates to assume these roles [16, 17].

Reducing postfellowship barriers to practicing ID could expand the pool of applicants for ID fellowships and offer the ID community an opportunity to advocate for support from national organizations and state and federal governments. For internal medicine residency graduates, compensation for hospitalist jobs is usually more than ID physician salaries, and work to close this gap could steer interested candidates to fellowship [18]. Comprehensive loan repayment programs, some of which are in pilot stages, could expand to encourage recruitment, particularly in areas of ID shortages [19]. For non-US citizen international medical graduates, increasing H-1B visa sponsorship would make transitioning into an ID career easier. For those with J-1 visas, increasing waiver opportunities on graduation, including opportunities to provide ID telemedicine to underserved areas, could increase the ID workforce. Furthermore, for international medical graduates who have completed an international residency, removing the onerous barrier of additional US residency training for board certification could bring qualified candidates to ID [20]. A Pilot Model Pathway proposed by the American Board of Internal Medicine Council is under review.

## POSITIVE MESSAGING

Although challenges to workforce development exist, ID specialists can positively impact how the field is perceived. Celebrating and promoting the value, importance, and accomplishments of the ID specialty through publications, program websites, and social media, can inspire the next generation of physicians to join our diverse, rich community. Recruitment efforts should showcase the breadth of opportunities available within an ID career and the flexibility and personal fulfillment it offers members (Table 2). Trainees should hear how ID physicians value their quality of life and have high levels of professional satisfaction and flexible schedules. ID physicians providing direct patient care find a deep reward as they help to cure, control, and prevent infections, including those occurring in the most vulnerable populations. These meaningful and rewarding aspects of a career in ID should be highlighted with trainees as they consider ways to contribute to this valuable work.

All ID providers are encouraged to serve as ambassadors of the specialty by creating individual connections and serving as mentors to students, residents, and other trainees. These connections can be local or through IDSA programs connecting mentors with mentees across the country. Intentional efforts should be made to highlight the diversity of the community so that trainees feel welcome and represented. At the same time, issues of workload and undercompensation demand continued attention, and efforts to address these concerns with real solutions should be highlighted.

## CONCLUSION

ID professionals should be intentional in the connections they build with trainees and in the messaging they put forth, to showcase the breadth, depth, and appeal of the field. Advocating for and supporting programs designed to foster a robust, resilient ID community can ensure the future of this exciting, ever-changing, and indispensable field of medicine.
